# Effect of using artificial intelligence chatbot about electronic fetal monitoring on maternity nursing students’ performance

**DOI:** 10.1186/s12909-025-08391-1

**Published:** 2025-12-18

**Authors:** Amal Mohamed Talaat Abdelwahab, Marwa Ibrahim Hamdy Aboraiah, Hanan Elsayed Mohamed Elsayed

**Affiliations:** Department of Woman’s Health and Midwifery Nursing, Faculty of Nursing, Mansoura University, Mansoura , Egypt

**Keywords:** Chatbot, Education, Electronic fetal monitoring, Artificial intelligence

## Abstract

**Background:**

The integration of artificial intelligence chatbots in education has led to numerous possibilities, providing a focused, personalized, and result-oriented learning environment that enhances students’ cognitive and interpretive skills. Electronic fetal monitoring-related tasks require professional knowledge and clinical insight; with the aim of supporting students in making accurate decisions when dealing with real cases, it is necessary to engage them in authentic problem-solving contexts.

**Aim of the study:**

This study aims to evaluate the effectiveness of an electronic fetal monitoring (EFM) chatbot in improving maternity nursing students’ theoretical knowledge, practical interpretation skills, confidence in clinical reasoning, academic motivation, and satisfaction with feedback.

**Methods:**

A quasi-experimental design was conducted among 84 undergraduate nursing students at the Faculty of Nursing, Mansoura University, Egypt, allocated into an intervention group (*n* = 42), received artificial intelligence chatbot–based education, and a control group (*n* = 42), received traditional teaching method. Data were collected using: (1) a self-administered questionnaire to assess students’ knowledge, interpretation skills, and clinical reasoning confidence regarding electronic fetal monitoring; (2) the Academic Motivation Scale; and (3) a students’ satisfaction survey. Data were analyzed with SPSS v22.0. Normality and homogeneity of variance were assessed with the Shapiro–Wilk and Levene’s tests, respectively. Continuous variables were reported as means and standard deviations, and categorical variables as numbers and percentages. Chi-square, Fisher’s exact, and paired t-tests were employed for group comparisons as appropriate. Effect sizes were estimated using Cohen’s d and Eta squared (η²). Statistical significance was set at *p* < 0.05.

**Results:**

Compared with the control group, the current research revealed statistically significant improvements in students’ knowledge, practical interpretation, and critical reasoning regarding fetal monitoring, as well as academic motivation among students who received chatbot education at the post and follow-up tests (*p* < 0.001). In addition, 90.5% of the participants in the intervention group reported high levels of satisfaction with the chatbot.

**Conclusion:**

Chatbot education significantly improved students’ theoretical knowledge, practical interpretation skills, confidence in clinical reasoning, and academic motivation, with sustained gains observed at both the post and follow-up tests. Additionally, students reported high satisfaction with the feedback provided by the chatbot.

**Trial registration:**

ClinicalTrials.gov, NCT07051343, registered on June 6, 2025.

**Supplementary Information:**

The online version contains supplementary material available at 10.1186/s12909-025-08391-1.

## Introduction

Recent advances in information and communication technology have greatly reshaped the landscape of education. In parallel with these advancements, artificial intelligence (AI) has emerged as a primary area of interest. AI imitates human intelligence processes via machines to maximize their chances of achieving a specific goal [1–3].

In recent years, the integration of AI into education has become increasingly prevalent, leading to numerous possibilities, such as an intelligent tutor, a collaborative learning partner, and a provider of a focused, personalized, and results-oriented online learning environment, benefiting both educators and students [4, 5]. Among the different types of AI applications, chatbots represent one of the most commonly encountered AI-based tools in the education field [6].

Chatbots refer to automated conversational or interactive agents that use natural language processing and machine learning algorithms to mimic and process human communication, enabling people to interact with digital devices as if they were speaking with a real person [4]. The effective incorporation of chatbots into education and training can positively affect the academic success of students [7]. The compatibility of chatbots with course content, their flexible structure, suitable design, creative and supportive features, and attractiveness enhance their efficiency in the learning process [8]. Considering the benefits that chatbots can offer within the scope of education and training, it is crucial to integrate this software into nursing education-training activities [9].

Nursing education and training aims include not only mastering skills but also developing decision making abilities for effective problem solving [10]. With respect to essential nursing practices in midwifery nursing, education about the installation of electronic fetal monitoring (EFM) equipment and the interpretation of its results is needed [11]. EFM is a method to assess fetal health by recording fetal heart rate and uterine contraction or fetal movements utilized to detect signs of fetal distress and provide interventions at an early stage by identifying changes in fetal heart patterns, thus minimizing antepartum or intrapartum fetal compromise [12]. Approximately one-third of global neonatal mortality each year is attributed to intrapartum-related complications, including birth asphyxia [13].

Within the health sciences domain, the majority of training programs rely on a lecture-based teaching approach, which offers students limited opportunities for deep engagement with subject matter due to the minimal interaction and lack of contextual learning. This limitation may negatively affect their learning efficacy and clinical judgment [10]. Since EFM-related tasks demand both professional knowledge and clinical insight, ensuring that nursing students receive adequate education and training in EFM prior to their clinical placement in delivery settings is essential. To support students in making accurate decisions when dealing with real cases, it is necessary to engage them in realistic, problem-based contexts that mirror clinical practice [10, 11].

Accordingly, the integration of an AI-based educational chatbot may serve as an effective pedagogical tool to enhance students’ cognitive and interpretive skills in EFM. This AI-supported approach allows for guided, repetitive practice in a risk-free environment, thereby strengthening clinical decision-making competence before real clinical application.

### Significance of the study

Conventional education systems are challenged by several issues, such as overcrowded classrooms, high student-to-teacher ratios, limited personalized student attention, and disparities in learners’ pace and style [14]. Consequently, these factors often contribute to decreased student satisfaction and subsequently, hinder learning outcomes and overall educational effectiveness [15]. Based on Egypt Vision 2030, one of the challenges in the health science sector is the mismatch of skills between higher education graduates and the needs of the health sector [16]. Given that the priority agenda of the Egyptian government is reducing the Maternal and Neonatal Mortality Rate and increasing life expectancy at birth to 31% and 75%, respectively, the existing mismatch between the competencies of health-science graduates and the evolving needs of the health sector highlights a gap in the national workforce [17].

To further bridge the gap between theoretical knowledge and practical application at the university level, the Egyptian Ministry of Communications and Information Technology, in partnership with the Ministry of Higher Education and Scientific Research, has initiated the implementation of AI technologies in the university educational infrastructure. This initiative has encouraged Egypt to reach its sustainable developmental goals (SDGs) [18, 19].

In this context, it is important to examine the effects of chatbots, which have the potential to be used in educational institutions that have the mission of shaping society and guiding the future of all education stakeholders. As such, there is a growing need to design and evaluate chatbot-based programs within nursing education in Egypt. This study was undertaken to address this gap and contribute to the evidence base supporting the integration of AI in educational contexts.

### Aim of the study

The current research aimed to evaluate the effectiveness of an electronic fetal monitoring (EFM) chatbot in improving maternity nursing students’ theoretical knowledge, practical interpretation skills, confidence in clinical reasoning, academic motivation, and satisfaction with feedback.

### Research hypotheses

To accomplish the study’s aim, the following hypotheses were developed:

- H1. Maternity nursing students who received EFM chatbot education will have better theoretical knowledge regarding EFM than those in the control group.
- H2. Maternity nursing students who received EFM chatbot education will have satisfactory practical interpretation skills regarding EFM compared to those in the control group.
- H3. Compared with the control group maternity nursing students who received EFM chatbot education will have more confidence in their clinical reasoning regarding EFM.
- H4. Maternity nursing students who received EFM chatbot education will have increased academic motivation compared to the control group.
- H5**.** Maternity nursing students who received EFM chatbot education will have high satisfaction with the feedback.

## Method

### Research design

To accomplish the aim of this study a quasi-experimental, pretestـposttest control group design was employed. The study followed the guidelines provided in the TREND Statement checklist [20]. Additionally, the study protocol was formally registered on ClinicalTrials.gov (identifier code: NCT07051343) registered on June 6, 2025.

### Study subjects

**Fig. 1:**
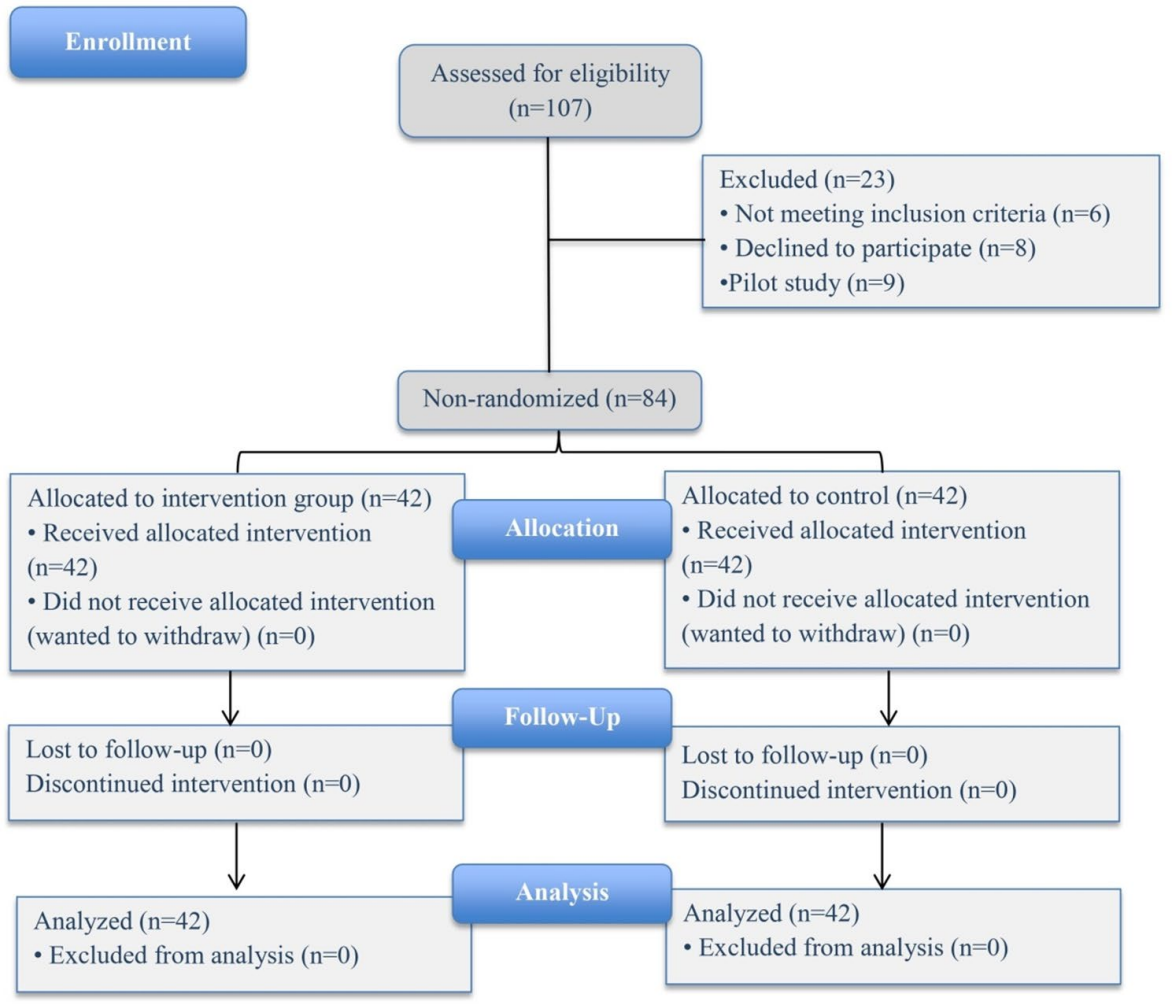
Flow chart of the study participants

The subjects were recruited via purposive sampling from third-level students who were enrolled in a midwifery course during the 2024/2025 academic year at the Faculty of Nursing, Mansoura University, Egypt. The participants were recruited through multiple approaches. A formal announcement was made in coordination with the batch leader, briefly describing the study and its purposes and emphasizing the voluntary nature of participation. Additionally, to enhance accessibility and convenience, the researcher was physically present at the Women’s Health and Midwifery Nursing Department to provide study-related explanations and address participants’ inquiries, thereby fostering engagement and encouraging participation. Students who had no experience with EFM or EFM education via Chatbot were enrolled in the study. Among the 107 students initially approached for participation, 23 were excluded, with 6 not meeting the specified criteria, 8 declined to participate, and 9 were used for piloting purposes. Eventually, a total of 84 students were included in the study. The subjects were divided into an intervention group, which included 42 students who received the AI chatbot education and a control group, which included 42 students who received the traditional learning method [online meeting via Microsoft Teams] (Fig. 1).

### Study setting

The study was carried out at the Faculty of Nursing, Mansoura University, located in Dakahlia Governorate, which is affiliated with the Ministry of Higher Education, Egypt. The faculty is equipped with seven clinical laboratories with a capacity of 25–30 students/lab. The ratio of teaching assistants to students in the woman’s health & midwifery nursing department is 1:120.

### Sample size calculation

The following formula was employed to estimate the required sample size [21] on the basis of data from the literature [11], considering a power of 80% and, a level of significance of 5%, the sample size can be calculated using the following formula:

$$n\;=\;\:\frac{(\mathrm{Z}{\upalpha\:}/2\:+\:\mathrm{Z}{\upbeta\:})^2\:\times\:\:2\left(\mathrm{S}\mathrm{D}\right)^2\:}{d^2}$$

Where, SD = the standard deviation obtained from a previous study; Z_α/2_, for 5% this is 1.96; Z_β_, for 80% this is 0.84, and d, for the expected difference. Therefore,

$$n\;=\:\frac{(1.96\:+\:0.84)^2\:\times\:\:2\left(2.77\right)^2\:}{\left(1.7\right)^2}\;=\;41.6$$

On the basis of the above formula, the sample size required is 42 in each group.

### Tools of data collection

Three tools were employed for data collection:

#### Tool I: Self-administered questionnaire

This tool was developed by the researcher following an extensive review of relevant national and international literature [11, 22, 23], and [24]. It consists of 4 parts:

##### Part (1): General Characteristics of Maternity Students

Gender, GPA, previous certificate & previous training in EFM.

##### Part (2): Maternity Students’ Knowledge regarding EFM

This part consists of 32 questions, 6 related to the concept and application of EFM, 12 related to baseline fetal heart patterns and variability, and 14 regarding episodic and periodic changes in fetal heart rate.

##### Scoring system of knowledge

Students’ knowledge was assessed using a binary scoring method: each correct answer was given a score of 1, while incorrect answer was given a score of 0. The total score for each knowledge domain was calculated by summing the number of correct responses and dividing it by the total number of items to obtain a mean score. The knowledge level was classified as follows: good knowledge was defined as 80% or higher (score ≥ 27), average knowledge was defined as 60% to 79% (score 20–26), poor knowledge was defined as 40% to 59% (score 13–19), and very poor knowledge was defined as less than 40% (score ≤ 12) [22]. The instrument’s reliability was Cronbach’s alpha = 0.903, indicating high reliability.

##### Part (3): Maternity Students’ clinical Interpretation Competency regarding EFM

This part contains different trace photos, with several questions intended to assess the respondents’ understanding of it. The traces were described as pictures, each containing questions (a total of 40 questions) to test the students’ ability to detect symptoms and signs of fetal deterioration, interpret abnormalities in fetal cardiac patterns, and prioritize and manage fetal health issues.

##### Scoring system of clinical interpretation competency

A score of 1 was given for each correct response and 0 for each incorrect one. The scores of the items were summed for each area of fetal trace interpretation, and the total score was divided by the number of items, resulting in a mean score. Interpretation of the fetal traces was considered satisfactory if the percent score exceeded 60% (score >24), as opposed to an unsatisfactory (≤ 24) interpretation scoring [22]. The instrument’s reliability was Cronbach’s alpha = 0.899, indicating high reliability.

##### Part (4): Maternity students’ confidence in clinical reasoning regarding fetal health assessment

This section included 10 statements designed to assess maternity students’ confidence in their ability to collect patient history, perform appropriate assessment techniques, and recognize abnormalities based on the gathered data. The responses were rated on a 5-point Likert scale, ranging from “not confident at all” (1 point) to “strongly confident” (5 points).

#### Scoring system of confidence in clinical reasoning regarding fetal health assessment

The scores assigned to each item were summed, and then divided by the total number of items, resulting in a mean score. The score range was stratified as follows: 20% -<35% (10–17 points) indicates beginning; 35%−60% (18–29 points) indicates developing, 61%−85% (30–41 points) indicates achieving, and a score above 86% (42–50) indicates exemplary [24]. The reliability of the instrument was Cronbach’s α = 0.902, indicating high reliability.

#### Tool II: academic motivation scale (AMS)

The AMS was originally developed by Vallerand in 1993. In this study the scale was adapted from Ongor & Uslusoy to assess three dimensions: intrinsic motivation (4 items), extrinsic motivation (9 items), and A-motivation (5 items). Each item was rated on a 5-point Likert scale, ranging from 1 (does not correspond at all) to 5 (corresponds totally) [25].

#### The scoring system for AMS

The scores for all the items were summed, and the total was divided by the number of items, to calculate a mean score. A score < 35% (≤ 31 points) indicates low motivation; a score 35%−57% (32–52 points) indicates average motivation; and a score above 57% (53–90 points) indicates good motivation [25, 26]. The tool’s reliability in Ongor & Uslusoy’s study was Cronbach’s α = 0.83, while in the current study it was 0.897, indicating high reliability.

#### Tool III: Students’ Satisfaction with the Feedback regarding EFM Chatbot

Developed by the researcher following a thorough review of the relevant literature [27–29], comprises 13 items measured via a 3-point Likert scale. The response options ranged from 1(not satisfied) to 3 (satisfied). The total score was calculated by summing all the responses, and dividing the sum by the number of items. A satisfaction score ≤ 49% (≤ 19 points) indicates low satisfaction. A score from 50% to 69% (20–27 points) indicates average satisfaction. A score of 70% or above (≥ 28 points) indicates high satisfaction [27]. The instrument’s reliability was Cronbach’s α = 0.901, indicating high reliability.

#### Validity and reliability of the tools

The instruments employed in this study were carefully translated from their original languages (Korean and Turkish) into English, adhering to the WHO guidelines on the translation and adaptation of instruments to maintain both linguistic accuracy and cultural relevance [30]. To ensure the suitability of the translated instruments for students enrolled in an English-medium curriculum, a systematic and rigorous translation process was undertaken. This included forward translation, evaluation by an expert panel, back-translation, pretesting, and cognitive interviewing. Each step was carefully implemented to maintain the maximum level of accuracy, clarity, and contextual relevance of the translated questionnaires.

The content validity of the tools was assessed with the support of an expert panel, including a specialist in medical statistics. Their feedback was incorporated to refine the tools accordingly. Based on the validation process, revisions were made to enhance item clarity and ensure optimal comprehension. Prior to finalizing the English versions, a pilot test was conducted with 9 students to identify and modify any items that may have caused confusion, ensuring that the tools were appropriately adapted to the specific linguistic and academic setting. The Cronbach’s α of the self-administered questionnaire (tool 1) was 0.973 for the total questionnaire and 0.903, 0.899, and 0.902 for the subparts. The reliability of the AMS (tool 2) was 0.897, and the satisfaction with the feedback regarding EFM Chatbot (tool 3) was 0.901.

### Ethical considerations

Ethical approval was obtained from the Research Ethics Committee of the Faculty of Nursing (IRB000000594-21/7/2024). This study complied with the ethical principles stated in the Declaration of Helsinki. Prior to participation, all the students were provided with a detailed explanation of the study’s aims and procedures. Informed written consent was obtained using a standardized form approved by the Research Ethics Committee. Participation was entirely voluntary, and the students were assured that their involvement or declining to participate would have no consequences. Moreover, they were informed that they could withdraw from the study at any point without facing any form of penalty. The confidentiality of the collected data was strictly maintained, and the information was used exclusively for research purposes aligned with the study objectives.

### Data collection process

From the start of February 2025 until mid-July 2025, data were collected in 3 stages.

### Preparatory stage

This phase took 2 months; the researcher conducted a comprehensive review of the literature to develop the scientific content related to EFM. Particular attention was given to aligning the educational material with international guidelines and recommendations issued by reliable professional bodies in the field, such as the American College of Obstetricians and Gynecologists (ACOG), Association of Women’s Health, Obstetric and Neonatal Nurses (AWHONN), and the National Institute for Health and Care Excellence (NICE), to ensure that the information provided to participants was evidence-based and professionally validated.

Simultaneously, data collection tools were designed and prepared to be used later with the study participants. In parallel, the researcher enrolled in multiple training courses related to graphic design, video editing, and programming (Python language), as the study involved the development of an educational chatbot about EFM, which required multimedia content creation and technical implementation since these areas were outside the researcher’s primary field of specialization.

### Design stage

This phase took approximately 2 months; the researcher designed the chatbot using Python programming language to guarantee flexibility, functionality, and ease of content integration. The educational content included within the chatbot comprised both written text and custom-developed 3D videos, aiming to improve student engagement and accommodate various learning styles. Despite variations in format and delivery method, the theoretical content provided through the chatbot was similar to that presented to the control group, hence matching consistency in the core educational material across both groups.

Upon the completion of the initial design, a committee of experts from Woman’s Health and Midwifery Nursing, Obstetrics & Gynecology Medicine, and Information Technology reviewed the content, visual materials, and data collection tools. Based on their feedback, all required modifications were made to ensure the materials’ clarity, accuracy, and suitability prior to implementation. A pilot study was subsequently carried out on 10% of the target group (9 students) to assess the usability, clarity, and efficiency of the chatbot and the relevance of related tools. Based on the findings of the pilot study, further refinements were applied to enhance the quality and applicability of the intervention prior to the main implementation phase.

### Intervention and evaluation stage

During the intervention stage, the participants were allocated into two groups: the intervention group, which received the educational content via the chatbot, and the control group, which was educated using a traditional method (online meeting). Before the intervention was implemented, both groups were instructed to review introductory materials about EFM (recorded video) on the faculty’s official e-learning platform. This ensured a standardized baseline (reference point) understanding of the subject matter. Following this orientation, a pretest was administered to both groups to assess their initial knowledge, interpretation, and clinical reasoning. Each group subsequently received similar theoretical content through different educational materials and delivery methods. The intervention group was involved with the material through the chatbot, which provided interactive and multimedia learning experiences. In contrast, the comparison group received the content through scheduled online meeting via Microsoft Teams. Upon completion of the intervention, a posttest was conducted to evaluate immediate learning outcomes. Additionally, a follow-up assessment was administered six weeks later to assess knowledge retention over time.

### Statistical analysis

All the statistical analyses were performed using SPSS version 22.0 for Windows (SPSS, Chicago, IL). The Shapiro-Wilk test was applied to evaluate the normality of the data distribution. Normally distributed continuous data are presented as mean & standard deviation. Categorical variables are expressed as numbers and percentages. Levene’s test was utilized to verify the equality of variances between groups, while comparisons of categorical variables were conducted using the chi-square test or Fisher’s exact test when applicable. Independent t-tests were employed to examine differences in continuous variables between the groups at various time intervals. Additionally, Cohen’s d was calculated to determine the effect size of the observed differences, and Eta squared (η²) was used to assess the proportion of variance attributed to the intervention in the repeated measures analysis. The reliability of the study’s questionnaires was computed. The cutoff point for statistical significance was *p* < 0.05.

## Results

Table 1 shows the general characteristics of the studied groups. Females represented 73.8% of the study sample. With respect to GPA, 60.7% of the participants had GPAs ranging from 3.5 to < 4.0 and 86.9% of the sample held a previous high school certificate. The highest proportion in the intervention group reported an average level of interest in the maternal subject, with 28.6%, whereas 31.0% in the control group were not interested. Notably, none of the participants in either group had received previous training in EFM. Additionally, 76.1% of the participants had no prior experience with chatbots. Statistical comparisons between the two groups revealed no significant differences across all the characteristics (*P* > 0.05), indicating baseline comparability.

**Table 1:** General characteristics of the studied maternity nursing students (*n* = 84)

			Intervention		Control		significance test	
			*n*	%	*n*	%	X^2^	*P*
Gender	Female		34	81.0	28	66.7		
	Male		8	19.0	14	33.3	2.217	0.136
GPA	< 2.5		0	0.0	1	2.4		
	2.5 : < 3.0		3	7.1	6	14.3		
	3.0 : < 3.5		9	21.4	14	33.3		
	3.5 : < 4.0		30	71.4	21	50.0		
	4.0		0	0.0	0	0.0	4.675	0.197
Previous educational certificate	High School		37	88.1	36	85.7		
	Technical Institute		5	11.9	6	14.3	0.105	0.746
Level of interest in maternal nursing subjects	Very uninterested		8	19.0	4	9.5		
	Not interested		5	11.9	13	31.0		
	Average		12	28.6	12	28.6		
	Interested		8	19.0	9	21.4		
	Very interested		9	21.4	4	9.5	6.871	0.143
Previous training in EFM	Yes		0	0.0	0	0.0		
	No		42	100.0	42	100.0	0.000	1.000
Previous experience with a chatbot		Yes	9	21.4	11	26.2		
		No	33	78.6	31	73.8	0.263	0.608

χ2: Chi-square test/Fisher’s exact test

Table 2 presents the comparison between both groups at the pre-intervention test; across all the variables, Levene’s test is non-significant (*p* > 0.05), which indicates that homogeneity of variance is assumed between both groups. The Shapiro-Wilk test indicated that the data were normally distributed (*p* > 0.05).

**Table 2:** Homogeneity test of the dependent variable between both groups (*n* = 84)

	Group	Mean ± SD	Levene’s Test	Shapiro-Wilk	Sig.
Knowledge	Int.	7.9 ± 1.8	0.779	0.972	0.386
	Cont.	7.7 ± 1.7		0.964	0.199
Interpretation competency	Int.	5.9 ± 1.7	0.735	0.955	0.096
	Cont.	5.6 ± 1.8		0.954	0.089
Clinical Reasoning Confidence	Int.	14.1 ± 1.8	0.731	0.955	0.098
	Cont.	13.9 ± 1.7		0.957	0.118
Academic motivation	Int.	25.0 ± 3.5	0.603	0.978	0.599
	Cont.	24.3 ± 3.8		0.977	0.531

*Int*. intervention, *Con*. Control, *SD* Standard deviation, *Sig *Significant at *P* ≤ 0.05

Table 3 presents the total knowledge scores regarding EFM between the study and control groups at three time points. There was no significant difference between the groups at the pretest stage (t = 0.488, *P* = 0.627). However, highly significant differences were observed in both the posttest (t = 14.289, *P* < 0.001) and follow-up (t = 12.002, *P* < 0.001) results in favor of the intervention group. The effect size values (0.841 post, 0.803 follow-up) and η² values (0.833, 0.789, respectively) reflect a large practical effect.

**Table 3:** Total knowledge scores of the studied maternity nursing students regarding electronic fetal monitoring (*n* = 84)

	Group		Mean ± SD	T–test/*P*	95% CI	η²	Effect size
Pretest		Int.	7.9 ± 1.8	T = 0.488 *P* = 0.627	0.190 ± 0.390	0.006	0.057
		Con.	7.7 ± 1.7				
Posttest		Int.	30.6 ± 3.1	T = 14.289 *P* < 0.001**	14.333 ± 1.003	0.833	0.841
		Con.	16.3 ± 5.7				
Follow-up test		Int.	31.2 ± 2.1	T = 12.002 *P* < 0.001**	10.952 ± 0.911	0.789	0.803
		Con.	20.3 ± 5.3				

*Int*. Intervention, *Con*. Control, *SD* Standard deviation, *CI* Confidence interval, *η*² Eta-square

*(P) significant at *P* ≤ 0.05 ** significant at* P* ≤ 0.001

Table 4 demonstrates no statistically significant difference between the groups in the pretest phase (t = 0.788, *P* = 0.433); however, statistically significant differences were observed in the intervention group at both the posttest (t = 12.595, *P* < 0.001**)** and follow-up (t = 15.286, *P* < 0.001). Additionally, large effect sizes (0.809, 0.856) and η² values (0.796, 0.849) reflect a large practical impact of the intervention on interpretation competency among the intervention group.

**Table 4:** Total clinical interpretation competency scores of the studied maternity nursing students regarding electronic fetal monitoring (*n* = 84)

	Group	Mean ± SD	T–test/*P*	95% CI	η²	Effect size
Pretest	Int.	5.9 ± 1.7	T = 0.788 *P* = 0.433	0.309 ± 0.392	0.014	0.085
	Con.	5.6 ± 1.8				
Posttest	Int.	28.3 ± 7.6	T = 12.595 *P* < 0.001**	17.309 ± 1.374	0.796	0.809
	Con.	11.0 ± 4.6				
Follow-up test	Int.	32.1 ± 4.0	T = 15.286 *P* < 0.001**	17.571 ± 1.149	0.849	0.856
	Con.	14.6 ± 6.3				

*Int*. Intervention, *Con*. Control, *SD* Standard deviation, *CI* Confidence interval, *η*² Eta-square

*(P) significant at*P*≤ 0.05 ** significant at *P* ≤ 0.001

Table 5 illustrates no significant difference at baseline (t = 0.430 *P* = 0.668), whereas highly significant improvements appeared post-intervention (t = 8.269, *P* < 0.001) and at follow-up (t = 9.182, *P* < 0.001) in the intervention group. Effect sizes (0.669 post, 0.701 follow-up) and η² values (0.625 and 0.664) indicate a large effect in enhancing the intervention group’s clinical reasoning ability, with a maintained effect over time.

**Table 5:** Total scores of confidence in clinical reasoning of the studied maternity nursing students regarding electronic fetal monitoring (*n* = 84)

	Group		Mean ± SD	T–test/*P*	95% CI	η²	Effect size
Pretest		Int.	14.1 ± 1.8	T = 0.430 *P* = 0.668	0.166 ± 0.387	0.007	0.057
		Con.	13.9 ± 1.7				
Posttest		Int.	37.5 ± 7.5	T = 8.269 *P* < 0.001**	11.738 ± 1.419	0.625	0.669
		Con.	25.8 ± 5.3				
Follow-up test		Int.	38.6 ± 6.2	T = 9.182 *P* < 0.001**	11.857 ± 1.291	0.664	0.701
		Con.	26.7 ± 5.9				

*Int*. Intervention, *Con*. Control, *SD* Standard deviation, *CI* Confidence interval, *η*² Eta-square

*(P) significant at *P*≤ 0.05 ** significant at *P*≤ 0.001

Table 6 shows no initial significant difference between the two groups (t = 1.023 *P* = 0.309), confirming group equivalence. Significant increases in motivation were found post-intervention (t = 10.004, *P* < 0.001) and at follow-up (t = 14.663, *P* < 0.001) in the intervention group. The η² values (0.707 posttest, 0.840 follow-up) and effect sizes (0.735, 0.848) point to a large effect of chatbot education on the academic motivation of the intervention group.

**Table 6:** Total academic motivation scores of the studied maternity nursing students regarding electronic fetal monitoring (*n* = 84)

	Group		Mean ± SD	T–test/*P*	95% CI	η²	Effect size
Pretest		Int.	25.0 ± 3.5	T = 1.023 *P* = 0.309	0.761 ± 0.744	0.018	0.095
		Con.	24.3 ± 3.8				
Posttest		Int.	71.6 ± 11.4	T = 10.004 *P* < 0.001**	20.261 ± 2.025	0.707	0.735
		Con.	51.4 ± 6.6				
Follow-up test		Int.	77.3 ± 6.9	T = 14.663 *P* < 0.001**	23.523 ± 1.604	0.840	0.848
		Con.	53.7 ± 7.8				

*Int*. Intervention, *Con*. Control, *SD* Standard deviation, *CI* Confidence interval, *η*² Eta-square

*(P) significant at *P* ≤ 0.05 ** significant at *P* ≤ 0.001

**Fig. 2:**
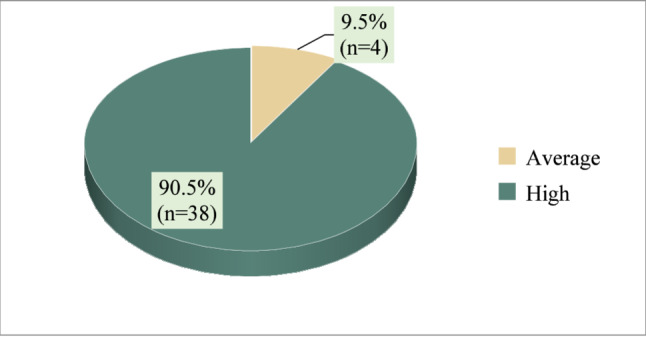
Total satisfaction with feedback of the intervention group regarding electronic fetal monitoring Chatbot (*n* = 42)

Figure 2 illustrates the satisfaction with feedback of the intervention group regarding electronic fetal monitoring Chatbot with a total of (90.5%) of the intervention group had a high level of satisfaction with the EFM chatbot, whereas **(9.5%)** had an average level of satisfaction.

## Discussion

The present study aimed to evaluate the effectiveness of an electronic fetal monitoring (EFM) chatbot in improving maternity nursing students’ theoretical knowledge, practical interpretation skills, confidence in clinical reasoning, academic motivation, and satisfaction with feedback. The findings demonstrated statistically significant improvements across all measured outcomes among students who received a chatbot education compared with those in the comparison group.

All participants were third-year undergraduates from the faculty of nursing; females were predominant in the study sample, whereas males represented less than one-third of the sample. Approximately two thirds of the participants reported a GPA of more than 3.5. The majority of the sample had a previous high school certificate, and nearly one third of the participants reported average interest in the maternity nursing specialty. Notably, none of the participants had previous knowledge or training regarding EFM. The homogeneity analysis between both groups using Levene’s test showed no significant differences. Additionally, the Shapiro-Wilk test was used to assess the normality of the data, and all outcome measures demonstrated a normal distribution, supporting the use of parametric tests.

With respect to students’ theoretical knowledge of EFM, the current research found no significant difference between the groups at the pretest stage, indicating the same baseline knowledge. However, the intervention group demonstrated significantly higher knowledge scores at both posttest and follow-up (*P* < 0.001), with large effect sizes suggesting a meaningful practical impact. These findings suggest that the chatbot appeared to enrich students’ understanding of EFM and may have supported immediate and long-term retention of knowledge through repeated, interactive engagement.

The higher knowledge scores achieved by the intervention group, as opposed to the control group may be due to the pedagogical advantages of chatbot-based learning, which provides students with instant, structured, and accessible educational content that can be revisited multiple times at their convenience, an essential factor known to enhance knowledge retention. In addition, the chatbot provided immediate feedback and clarification, helping students correct misunderstandings in real time and reinforcing accurate knowledge, which is difficult to achieve in conventional classroom settings with limited instructional time and larger student-to-instructor ratios.

This finding is consistent with a previous investigation in Taiwan that employed a chatbot for prenatal education and examined its effect on nursing students’ learning achievements. The study found that the chatbot-based learning strategy yielded better results and enhanced students’ learning outcomes [10]. Another study utilized (Termbot) an online chatbot-based learning model for medical terminology with 60 participants, and reported significant progress in learning outcomes in the intervention group [31].

In contrast, the findings differ from those of studies performed by “Han et al.”, who reported no significant difference in knowledge acquisition between the groups [11, 32]. Furthermore, an investigation by Shin et al. into AI-assisted learning (ChatGPT version 3.5- OpenAI) among 99 pediatric nursing students demonstrated significant group differences, with the control group exhibiting higher mean ranks [33].

Concerning practical interpretation skills, although the students in both groups were instructed to review introductory materials about EFM on the faculty’s e-learning platform prior to the pretest, many of them were unable to correctly answer the interpretation competency questions. This low rate of correct responses may be attributed to the nature of the introductory content, which likely focused on theoretical aspects rather than the interpretative application of fetal heart rate recordings. Practical interpretation skill is a higher-order clinical skill that requires exposure to real or simulated cases, and pattern recognition; thus without guided examples, feedback, or visual practice, students are unable to transfer theoretical knowledge to applied interpretive performance.

Following the intervention, participants in the chatbot group showed statistically significant improvement at both measurement points (*P* < 0.001), with high effect sizes compared with those in the control group. These results indicate that the intervention not only enhanced students’ immediate interpretive ability but also reinforced their analytical skills over time. This improvement may be attributed to the microlearning strategy of the designed bot, whereby educational content is divided into manageable parts, facilitating better comprehension and minimizing cognitive overload. Additionally, the visual and repetitive nature of chatbot-based case scenarios likely promoted pattern recognition and supported the interpretive skills of FHR tracings. Contrary to the findings of the present study, a quasi-experimental study in Korea reported that the critical analysis skills of nursing students in the comparison group who relied on textbooks were greater than those in the AI-education group [33].

Regarding students’ confidence in their clinical reasoning in the fetal health assessment, the pretest scores were the same, with no significant differences between the groups. The scores in the intervention group significantly increased after the intervention and during follow-up (*P* < 0.001), indicating a large effect size. These results indicated that the chatbot enhanced clinical reasoning by providing intricate case-based scenarios demanding active decision-making; this finding suggests that chatbots may serve not only as knowledge tools but also as platforms to support cognitive training using case-based scenarios in a less threatening environment. In addition, the chatbot’s adaptability and accessibility formed a learner-centered atmosphere that probably contributed to enhancing students’ engagement and greater critical thinking skills among the intervention group.

This finding is in line with previous research examining the use of chatbot-enhanced education among fourth-year nursing students, which revealed that the experimental group exhibited significantly greater clinical reasoning competency than did the control group [32]. Similarly, in Turkey, Arkan et al. assessed the effect of training using ChatGPT in nursing process implementation on undergraduate students’ clinical reasoning and problem-solving abilities. Their findings indicated a notable improvement in reasoning competency within the experimental group following the training, whereas the control group showed no significant changes [34].

Nevertheless, Baglivo et al. who conducted a study in Italy among 5th year medical students and utilized five AI Chatbot models (Bing Chat, ChatGPT, Chatsonic, Google Bard, and YouChat) & Abd-Alrazaq et al., contradict, as they argued that chatbots are not reliable substitutes for clinical reasoning in medical education, citing their inconsistent performance and the risk of producing inaccurate or misleading content [35, 36]. Similarly, another investigation in Korea reported that the introduction of a chatbot-based educational intervention among nursing students did not lead to a statistically significant improvement in clinical reasoning compared with the control group [11].

Pertaining to students’ academic motivation, the results indicated no significant difference in motivation at baseline. However, statistically significant increases were observed in the intervention group at both the posttest and follow-up stages (*P* < 0.001). The results showed a high effect size, suggesting a sustained motivational impact. The significant improvement in academic motivation among the intervention group can be explained by the interactive and personalized nature of AI-based learning which promotes active engagement, immediate feedback, and on-demand access to learning materials, unlike traditional teaching methods, which often rely on passive information delivery.

Besides, the features of the chatbot align with the core concept of self-determination theory, which emphasizes that learners are more motivated when they perceive autonomy, competence, and connection. By enabling students to regulate their own learning pace and revisit materials as necessary, the chatbot fostered a sense of independence and engagement, ultimately supporting the development of intrinsic motivation. Moreover, the conversational style of interaction may have reduced performance anxiety, increased students’ curiosity, and made the learning experience more engaging, especially for students who may struggle in traditional lecture-based environments, which are known to influence academic motivation positively.

In line with this finding, Chang et al., reported that, compared with traditional lecture-based method, the use of mobile chatbot in nursing training improved students’ interest in learning [10]. Additionally, a recent scoping review highlighted that chatbots designed based on motivational frameworks, such as self-determination theory, may contribute to greater student engagement and sustained learning efforts. Furthermore, studies by Hmoud et al. and Yilmaz & Yilmaz confirmed that AI-based learning tools significantly increase intrinsic motivation across educational courses [37, 38].

Conversely, various sources and publications have expressed concerns about the potential for AI to reduce cognitive effort and long-term motivation. For example, a study in China conducted on 117 university students from a diverse array of disciplines described “metacognitive laziness” resulting from overreliance on AI, where learners showed reduced engagement and ownership of their work [39]. In addition, Hong et al., employed a microlearning chatbot (A-MINC) as an instructional tool for 49 nursing students and reported no statistically significant differences in academic efficacy between the intervention and control groups [40].

Beyond academic motivation AI-based tools have also been found to foster higher-level skills. For instance, Agaoglu et al. reported that AI usage, when combined with digital literacy and academic support, enhanced students’ creative thinking [41]. Also, Tarsuslu et al. demonstrated that digital leadership could reduce anxiety and positively shape students’ attitudes [42]. Cross-national evidence further underscores the broader implications of AI chatbot use in education. A large-scale study involving 550 nursing students from different countries revealed that AI chatbot usage was positively associated with academic performance, with technophobia and technophilia acting as mediating factors. Specifically, technophobia weakened the positive impact of chatbot use, whereas technophilia amplified its beneficial effects [43]. These findings emphasize that, in addition to knowledge, reasoning, and motivation, psychological orientations toward technology such as technology-related fears and enthusiasm can shape the effectiveness of chatbot-mediated learning.

Similarly, a recent scoping review synthesized findings from multiple studies on the use of AI-based chatbots in nursing education. The analysis revealed not only improvements in students’ cognitive outcomes but also positive affective and behavioral effects, including greater satisfaction and active engagement. However, it also identified challenges related to chatbot design quality and faculty readiness for implementation [44]. Consistent evidence has also emerged across different disciplines and learner populations. For example, a survey conducted among 286 Austrian students examined the educational impact of AI-based chatbots on self-reflection and metacognitive regulation, reported significant improvements in students’ self-regulation and confidence, along with reduced learning anxiety and deeper learning engagement [45]. Furthermore, a quasi-experimental study among Saudi students found significant improvements in both critical thinking and self-regulated learning among those who used an AI-based educational chatbot. In addition, higher levels of AI literacy were shown to significantly predict students’ perceived value and intention to use chatbots as learning tools, suggesting that familiarity with AI technologies can influence learners’ motivation and willingness to adopt chatbot-assisted education [46].

With respect to students’ satisfaction with the feedback regarding the EFM chatbot, the majority of the experimental group had a high level of satisfaction, indicating positive acceptance and perceived value of chatbot-based learning. This finding is in agreement with that of Arkan et al. & Baglivo et al., who reported notably high satisfaction levels among students in the intervention group who engaged with chatbot education, reporting a score of 7.9 on a 10-point Likert scale. The participants highlighted the innovative and interactive nature of the chatbot, noting that it brought a refreshing enhancement to conventional instructional methods and expressed interest in repeating the experience [34, 35]. On the other hand, previous quasi-experimental studies evaluated feedback using a two group design (Chatbot vs. traditional lectures) and reported no significant differences between the two approaches. Although their analytical framework differs from the single group feedback assessment used in the current study, their findings provide a conceptual context suggesting that chatbot-based instruction is at least comparable to traditional instruction [11, 32].

In addition to the findings of the present study, AI interventions have been associated with broader outcomes as Pérez-Marín reviewed a wide range of pedagogic conversational agents that have been implemented in both school and university contexts. Notable examples include AutoTutor, which supports deep reasoning through dialog; Andes Physics Tutor and Conceptual Physics Tutor, which are designed to enhance problem-solving in science domains; A.L.I.C.E., and Oscar, which are adapted as text-based conversational agents for language learning; and Sam Math Tutor, which is used to strengthen mathematical problem-solving skills. These chatbots have consistently been shown to improve learners’ motivation, comprehension, and higher-order thinking across diverse student populations [47]. In the domain of language learning, several studies have reported that generative AI chatbots can alleviate students’ anxiety, improve communication competence, and foster positive attitudes toward interactive and accessible formats. Moreover, a mixed-methods study demonstrated that self-developed chatbots used as personalized writing assistants enhanced learners’ writing motivation and performance [48–51]. Extending beyond language education, research in engineering has shown that chatbots can foster self-directed learning, facilitate understanding of complex programming concepts, and improve the overall quality of student work [52].

Overall, AI chatbots have shown promising outcomes across diverse educational disciplines; however, their effectiveness relies on sound instructional design, contextual appropriateness, and the degree of learner engagement they foster. Accordingly, careful alignment with defined learning objectives and users’ educational needs is essential to ensure their meaningful and sustainable integration into teaching and learning processes.

## Conclusion

The outcomes of this research suggest that AI-driven chatbot educational program may enhance maternity nursing students’ performance related to EFM. In this study the intervention was associated with improvements in students’ theoretical knowledge, practical interpretation skills, confidence in clinical reasoning, and academic motivation, with positive effects observed at both the posttest and follow-up stages. These gains were supported by large effect sizes, indicating both statistical significance and potential educational relevance. Furthermore, the high levels of satisfaction with the feedback reported by the students in the intervention group suggested that the chatbot was positively received, enhancing user engagement and the perceived value of the learning experience.

### Recommendations

In light of the study findings, the following is recommended:

- Provide structured training programs for academic staff to increase their use of AI tools in educational contexts.
- Equip academic members with the essential skills to effectively design, implement, and critically assess AI-integrated teaching strategies.
- Broaden the use of AI-driven chatbots across nursing and other health-related disciplines.
- Pilot and expand AI-based teaching tools within nursing and allied health programs.

These recommendations are aligned with successful initiatives implemented at Bethlehem University which introduced an AI training program for more than 300 staff members to strengthen their capacity to use AI tools in teaching, research, and administration [53]. Likewise, the University of Alabama established an AI taskforce and developed a graduate-level certificate program to integrate AI into nursing curricula [54]. At the international level, programs such as the AI Faculty Development Program at the Innova Training Institute (Dubai) and the AI in Education Initiative at International Hellenic University (Greece) demonstrate institutional investment in equipping academic staff in designing, implementing, and critically evaluating AI-enhanced educational practices [55, 56]. Collectively, these examples illustrate the practicality of the abovementioned recommendations and offer transferable models that could be adapted to advance nursing education in Egypt.

### Further studies

- Investigate learners’ perceptions and engagement factors in AI-based education.
- Examine the effect of customizable chatbot education on cognitive outcomes.
- Comparative effectiveness of chatbot-based learning vs. high-fidelity simulation in skill acquisition.
- Barriers and facilitators to implementing AI chatbots in clinical nursing education: perspectives from educators and students.

### Study limitations

One of the limitations of this study is that it was conducted at a single university and included only third-year nursing students, which may restrict the generalizability of the findings. The sample size limits the extent to which the findings can be extrapolated. In addition, student satisfaction was assessed exclusively through a Numerical Rating Scale (NRS). While the NRS is a widely employed tool in both educational and clinical contexts due to its simplicity, it may not capture the depth of students’ experiences as effectively as qualitative approaches. Future investigations are encouraged to recruit larger and more diverse samples, spanning students from different academic years and institutions, and to employ mixed-methods approaches that integrate both quantitative and qualitative data. Accordingly, although the current findings are promising, future researches are needed to validate and expand upon these results in broader educational contexts. Nonetheless, we hope that the current study offers a reference point for further research exploring AI-based learning tools among Egyptian nursing students.

### Practical implications

The findings of this study demonstrate that AI-based chatbots can serve as effective supplementary tools in nursing education, enhancing students’ knowledge, interpretation, and clinical reasoning while promoting motivation and engagement. By providing instant, personalized feedback, chatbots help bridge the gap between theoretical learning and clinical practice, supporting reflective decision-making during training. This approach also facilitates continuous learning beyond classroom hours, making it suitable for blended and remote education models. Furthermore, adapting chatbot content to other areas or specialties may extend its educational value. From an institutional perspective, integrating AI-driven learning tools can reduce faculty workload, improve student engagement, and offer scalable solutions for resource-limited settings where access to simulation labs or clinical placements is restricted. These outcomes highlight the value of investing in AI-enhanced learning infrastructures to strengthen nursing education, promote self-directed learning, and prepare future nurses for technology-driven healthcare environments.

## Supplementary Information

Supplementary Material 1.

## Data Availability

The datasets used and analyzed during the current research are available from the corresponding author upon rational request.

## Abbreviations

AI: Artificial Intelligence
EFM: Electronic Fetal Monitoring
KPIs: Key Performance Indicators
SDGs: Sustainable Developmental Goals
FHR: Fetal Heart Rate
AMS: Academic Motivation Scale
ACOG: American College of Obstetricians and Gynecologists
AWHONN: Association of Women’s Health, Obstetric and Neonatal Nurses
NICE: National Institute for Health and Care Excellence
NRS: Numerical Rating Scale
