# Supplementary figures and images for: Effect of using artificial intelligence chatbot about electronic fetal monitoring on maternity nursing students’ performance
Source: BMC Med Educ. 2025 Dec 18;26:120. doi: 10.1186/s12909-025-08391-1 (PMC12825235; doi:10.1186/s12909-025-08391-1)

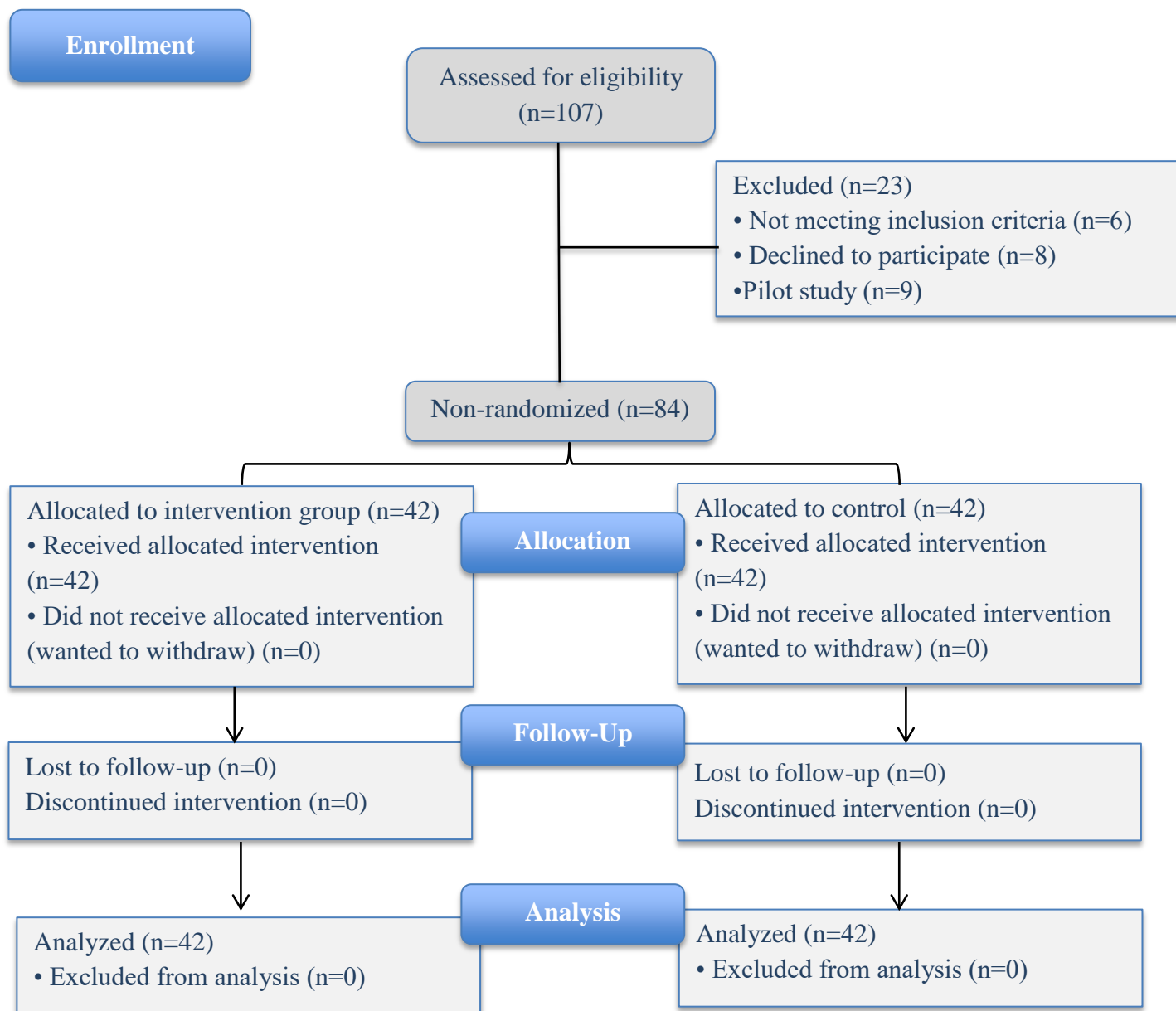

Supplement: Supplementary file 1 — Supplementary Material 1. [file 12909_2025_8391_MOESM1_ESM.pdf]
